# Combined parametric mapping allows discrimination of disease activity in myocarditis

**DOI:** 10.1186/1532-429X-18-S1-P256

**Published:** 2016-01-27

**Authors:** Johannes Schüler, André Rudolph, Luisa M Schmacht, Florian von Knobelsdorff-Brenkenhoff, Matthias A Dieringer, Andreas Greiser, Peter Kellman, Marcel Prothmann, Jeanette Schulz-Menger

**Affiliations:** 1 Charité Medical Faculty and HELIOS clinics, Working group Cardiovascular MRI, Berlin, Germany; 2 Siemens Healthcare, Erlangen, Germany; 3 grid.94365.3d0000000122975165Laboratory of Cardiac Energetics, National Institutes of Health/NHLBI, Bethesda, MD USA

## Background

Noninvasive detection of myocarditis is a unique feature of Cardiovascular Magnetic Resonance (CMR), but conventional CMR-imaging suffers from different challenges including imaging quality. It has already been shown, that T1 mapping has the potential to identify acute myocarditis [1,2].

We aim to differentiate the course of disease and to identify the acute stage by combining mapping techniques.

## Methods

Eighteen patients with clinically defined acute myocarditis were prospectively examined three times during follow up using a 1.5 T Scanner (FU0: within 2 weeks of symptoms onset, FU1: 5 weeks FU2: after 6 months). They were compared to a healthy sex- and age matched control group. Severity of disease was rated in NYHA and CCS score. Left ventricular (LV) volumes, mass and function were assessed using steady-state free precession (SSFP) cine imaging in short axis. Parametric mapping was applied using T2 maps in three short axis (TE 1.15 ms, ST 6 mm, SSFP) and native T1 mapping using modified look-locker inversion recovery (MOLLI 5(3)5) sequence (TE 1.11 ms, ST 6 mm) in a basal and a midventricular slice. Furthermore, T1 mapping was performed during contrast media wash-out (Gadobutrol 0.15 mmol/kgBW) at minute 1,3,5,7,10. Late gadolinium enhancement (LGE) was performed after minute 12 for myocardial fibrosis detection. Blood collection was done for haematocrit assessment. Data were analyzed using CVI42 (circle cardiovascular imaging Inc., Calgary, Canada), Mann-Whitney U test was used.

## Results

All 18 patients and healthy volunteers completed all examinations (age 30.06 ± 10.9 vs. 29.72 ± 9.93 years, 14 male and 4 female, respectively). NYHA score decreased from 1.8 ± 0.95 (FU0) to 0.5 ± 0.5 (FU2), CCS score decreased from 2.3 (FU0) to 0.3 (FU2).

14(78%) patients showed focal fibrosis (LGE pos).

Global T2 relaxation times decreased during follow up (p < 0.001 from FU0 to FU2). ROC analysis revealed a area under the curve of 0.9 for cutoff 51.92 ms (sensitivity 89%, specifity 83%) to differentiate diseased from healthy volunteers. Global native T1 remains stable (FU0 to FU2, p = 0.311), however, native T1 was significantly higher in patients than in healthy volunteers (p=0.003). Difference in T1 during contrast media washout between patients and healthy volunteers was significant at minute 3 at FU0 (321 ± 37 ms vs. 361 ± 31 ms, p = 0.001). Global ECV was increased at FU0 (p=0.021) and normalized at FU2 (p=0.164) compared to control group. See table 1 for details.

**Table 1:** Time course of LV ejection fraction and parametric mapping during follow up compared to healthy control group

	FU 0 acute phase	FU1 5 weeks	FU2 6 months	Healthy control group
ejection fraction (%)	58.5 ± 6.7 (p=0.133)	60.37 ± 6.1 (p=0.548)	60.03 ± 4.4 (p=0.658)	61.32 ± 3.7
Global T2 time (ms)	55.1 ± 2,6 (p < 0.001)	52.2 ± 1.4 (p=0.012)	51.2 ± 2 (p=0.429)	50.6 ± 1.9
Native T1 time (ms)	1015 ± 36 (p=0.003)	1003 ± 27 (p=0.015)	1001 ± 31 (p=0.043)	978 ± 32
Global ECV	0.259 ± 0.02 (p=0.021)	0.258 ± 0.02 (p=0.019)	0.256 ± 0.03 (p=0.164)	0.243 ± 0.02

## Conclusions

T1 mapping allows the identification of diseased in comparison to healthy volunteers. Additional use of T2 mapping enables to detect disease acuity and so far to identify reversibility. Further follow up examinations would be necessary to identify its prognostic values. Parametric mapping may replace parts of conventional imaging techniques.

**Figure 1:**
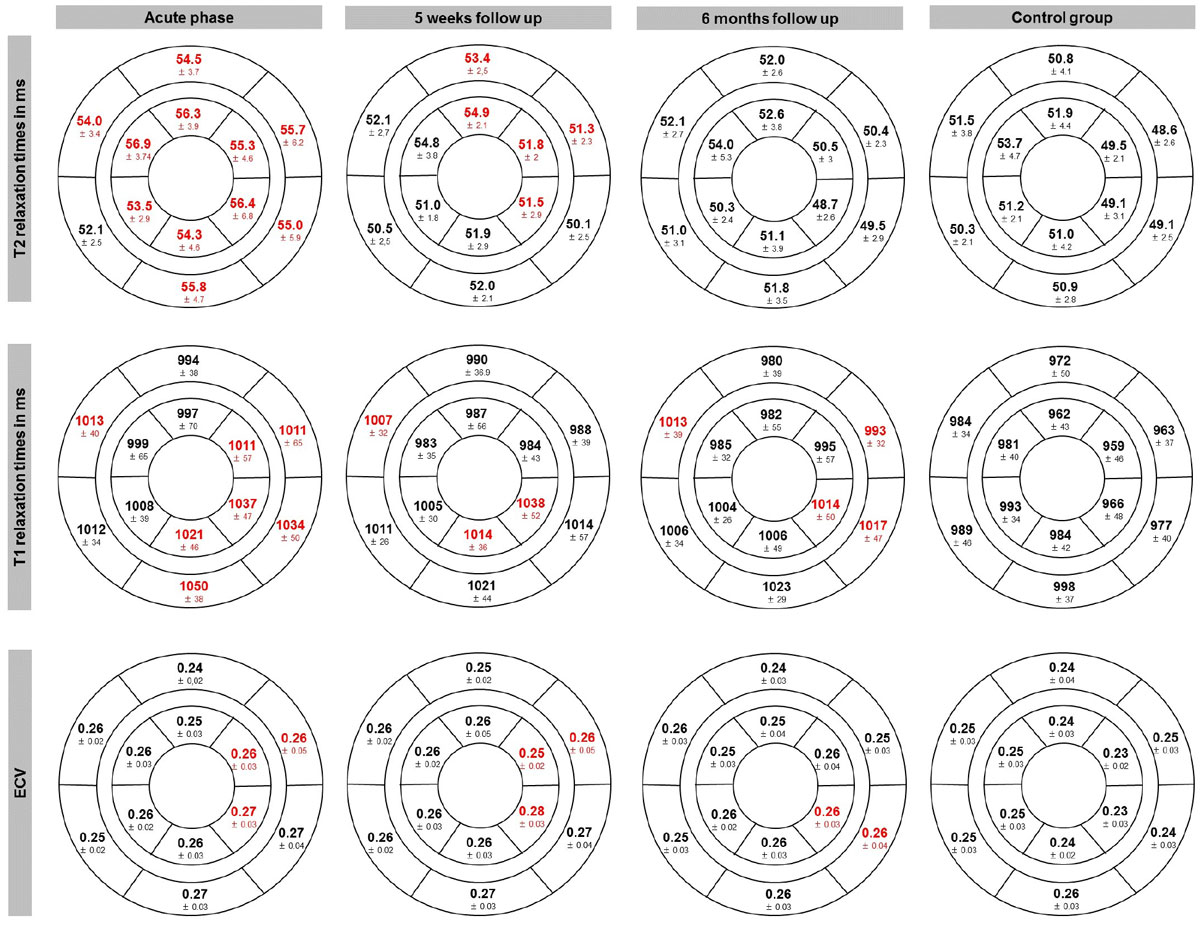
**Segmental T1, T2 times and ECV**. Red numbers mean that p < 0.05 compared to control group.
